# Community Arts Programming Supports Personal Growth and Well-Being in Older Adults

**DOI:** 10.1093/geroni/igaa057.3085

**Published:** 2020-12-16

**Authors:** Niyati Dhokai, Jatin Ambegaonkar

**Affiliations:** George Mason University, Manassas, Virginia, United States

## Abstract

Our research study examined how taking part in the arts compared to control affects older adults’ health and well-being. 64 older adults took part in dance, music, or control workshops 2 times/week for 10 weeks. We examined participants’ psychological health, social engagement, and personal growth outcomes using mixed methods during pre- and post-workshop assessments. Focus group and arts survey results revealed that participants felt ownership of new skills learned and felt engaged. Participants, especially for those in arts workshops, described having increased self-perception of creative skills resulting in a sense of personal growth, which occurred despite mind/body challenges experienced during workshops including musculoskeletal challenges, hearing impairments, and challenges retaining new information. Our observations provide avenues for future researchers to create programming that empowers older adults, as well as to utilize the participants’ ongoing feedback to create participant-empowered adaptations that transcends mind/body limitations through accessible pedagogical methods.

